# Inhibition of Mitochondrial p53 Accumulation by PFT-μ Prevents Cisplatin-Induced Peripheral Neuropathy

**DOI:** 10.3389/fnmol.2017.00108

**Published:** 2017-04-18

**Authors:** Magdalena A. Maj, Jiacheng Ma, Karen N. Krukowski, Annemieke Kavelaars, Cobi J. Heijnen

**Affiliations:** Laboratory of Neuroimmunology, Division of Internal Medicine, Department of Symptom Research, The University of Texas MD Anderson Cancer CenterHouston, TX, USA

**Keywords:** p53, mitochondria, cisplatin, peripheral neuropathy, pifithrin-μ, bioenergetics

## Abstract

Chemotherapy-induced peripheral neuropathy (CIPN), a debilitating major side effect of cancer treatment, is characterized by pain and sensory loss in hand and feet. Platinum-based chemotherapeutics like cisplatin frequently induce CIPN. The molecular mechanism underlying these neurotoxic symptoms is incompletely understood and there are no preventive or curative interventions. We hypothesized that cisplatin acts as a cellular stressor that triggers p53 accumulation at mitochondria, leading to mitochondrial dysfunction and CIPN. To test this hypothesis, we examined the effect of the small molecule pifithrin-μ (PFT-μ), an inhibitor of p53 mitochondrial association on CIPN and the associated mitochondrial dysfunction. We show here for the first time that *in vivo* cisplatin rapidly increases mitochondrial accumulation of p53 in dorsal root ganglia (DRG), spinal cord, and peripheral nerve without evidence for apoptosis. Cisplatin-treatment also reduced mitochondrial membrane potential and lead to abnormal mitochondrial morphology and impaired mitochondrial function in DRG neurons. Pre-treatment with PFT-μ prevented the early cisplatin-induced increase in mitochondrial p53 and the reduction in mitochondrial membrane potential. Inhibition of the early mitochondrial p53 accumulation by PFT-μ also prevented the abnormalities in mitochondrial morphology and mitochondrial bioenergetics (reduced oxygen consumption rate, maximum respiratory capacity, and adenosine triphosphate synthesis) that develop in DRG and peripheral nerve after cisplatin-treatment. Functionally, inhibition of mitochondrial p53 accumulation prevented the hallmarks of CIPN including mechanical allodynia, peripheral sensory loss (numbness) as quantified by an adhesive-removal task, and loss of intra-epidermal nerve fibers. In conclusion, PFT-μ is a potential neuroprotective agent that prevents cisplatin-induced mitochondrial dysfunction in DRG and peripheral nerves thereby protecting against CIPN through blockade of the early cisplatin-induced increase in mitochondrial p53. Notably, there is accumulating evidence that PFT-μ has anti-tumor activities and could therefore be an attractive candidate to prevent CIPN while promoting tumor cell death.

## Introduction

Chemotherapy-induced peripheral neuropathy (CIPN) characterized by pain, numbness, and tingling in hand and feet affects up to 70% of cancer patients during treatment but also persists into survivorship in a large number of patients (Seretny et al., 2014). Platinum-derived chemotherapeutics like cisplatin frequently cause CIPN and this represents a major cause of dose reduction.

Cisplatin-induced neuropathy in rats is associated with changes in nerve conduction, axonal degeneration, and nuclear DNA damage in dorsal root ganglia (DRG) (Podratz et al., 2011). It has been proposed that mitochondrial abnormalities and associated oxidative stress in peripheral sensory neurons contribute to chemotherapy-induced mechanical allodynia (Flatters and Bennett, 2006; Carozzi et al., 2010; Xiao et al., 2011; Areti et al., 2014). For example, in rats mechanical allodynia in response to paclitaxel or oxaliplatin treatment is associated with morphological and functional changes in mitochondria (Flatters and Bennett, 2006; Xiao et al., 2011). *In vitro* data obtained in primary cultures of DRG neurons indicate that cisplatin may cause early mitochondrial impairment with loss of membrane potential and induction of autophagy (Melli et al., 2008). However, it is not known if and how cisplatin treatment initiates mitochondrial damage *in vivo*, if and how this can be prevented and whether mitochondrial abnormalities are causally related to cisplatin-induced peripheral neuropathy.

The tumor-suppressor molecule p53 can promote mitochondrial damage via its activity as a transcription factor for pro-apoptotic proteins like p53 upregulated modulator of apoptosis (PUMA), Phorbol-12-Myristate-13-Acetate-Induced Protein 1 (PMAIP1 also known as Noxa), and Bcl-2 associated X-protein (Bax), all of which are involved in mitochondrial outer-membrane permeabilization and apoptosis. *In vitro* studies have shown that p53 also has transcription-independent effects involving translocation of p53 to mitochondria after cell stress (Marchenko et al., 2000). The *in vivo* relevance of mitochondrial p53 association is only beginning to be revealed. In a murine model of lethal irradiation, mitochondrial p53 translocation was detected in thymocytes within 30 min (Erster et al., 2004). We described a rapid association of p53 with brain mitochondria in a rodent model of ischemic brain damage and in response to cisplatin (Nijboer et al., 2011, 2013; Chiu et al., 2017).

The small compound pifithrin-μ (PFT-μ; 2-phenylethynesulfonamide) was identified as a compound that inhibits mitochondrial p53-accumulation without affecting the transcriptional activity of p53 (Strom et al., 2006). We showed that PFT-μ has a strong neuroprotective effect in a mouse model of neonatal ischemic brain damage (Nijboer et al., 2013). Furthermore, we recently demonstrated that PFT-μ protects against mechanical allodynia induced by paclitaxel and against cognitive deficits induced by cisplatin (Krukowski et al., 2015; Chiu et al., 2017).

Interestingly, in contrast to the neuroprotective effects, PFT-μ promotes tumor cell death via a mechanism involving protein aggregation, impaired autophagy, and inhibition of lysosomal function. This pro-apoptotic effect of PFT-μ appears to be specific for tumor cells and independent of p53. The existing data indicate that this pro-apoptotic effect of PFT-μ in tumor cells is mediated by disruption of the association between heat-shock protein 70 (HSP70) and its co-chaperones in proliferating cells with high levels of HPS70 (Leu et al., 2009).

In the present study we tested the hypothesis that cisplatin-induced peripheral neuropathy including allodynia, numbness, and retraction of intra-epidermal nerve endings, is caused by an early mitochondrial p53 accumulation in DRG neurons and peripheral nerve leading to an acute change in membrane polarization and subsequent long term mitochondrial dysfunction. To test this hypothesis we examined the effect of PFT-μ, an inhibitor of mitochondrial p53 accumulation on CIPN and the associated mitochondrial dysfunction. Most previous studies on CIPN in rodents have been performed in males while in humans pain is more prevalent in females. In addition, it has been shown that mitochondrial deficiencies develop in response to chemotherapy in male rodents (Xiao et al., 2011, 2012), but the effect of chemotherapy on mitochondrial function in females remains to be determined. Therefore, we performed our studies in female mice.

## Materials and methods

### Animals

Adult female C57BL/6 mice were group-housed at 22°C with a 12-h light/dark cycle (lights on at 6 am) and free access to food and water. All experimental procedures were performed according to the National Institute of Health Guidelines for the Care and Use of Laboratory Animals and the Ethical Issues of the International Association for the Study of Pain (Zimmermann, 1983) and were approved by the Institutional Animal Care and Use Committee of the University of Texas MD Anderson Cancer Center. All measures were performed by an investigator blinded to treatment.

### Drug administration

To induce CIPN, mice received two rounds of 5 daily i.p. injections of cisplatin (2.3 mg/kg/day) followed by 5 days of rest (cumulative dose 23 mg/kg) (Ta et al., 2010; Mao-Ying et al., 2014; Krukowski et al., 2015). Control mice were treated with saline according to the same schedule. In experiments on the biochemical analysis of the effects of cisplatin, mice received a single dose of cisplatin (2.3 mg/kg i.p) or saline. All cisplatin-contaminated items were discarded waste following Institutional Biosafety Committee guidelines.

PFT-μ (Sigma-Aldrich, St. Louis, MO, USA) was dissolved in dimethyl sulfoxide (25 mg/mL), diluted in sterile saline, and administered i.p. at a dose of 8 mg/kg 1 h prior cisplatin treatment (Nijboer et al., 2011; Krukowski et al., 2015).

### Mitochondrial isolation

DRG L2-L6, sciatic nerve, or spinal cord (L2-L6) (50–100 mg/sample) were homogenized in 100–400 μL of ice-cold buffer I (70 mM sucrose, 210 mM mannitol, 5 mM HEPES, 1 mM ethylenediaminetetraacetic acid (EDTA), and protease and phosphatase inhibitors) as described in Nijboer et al. (2007). Homogenates were incubated on ice for 30 min, centrifuged for 10 min at 800 *g*, 4°C, and followed by collection of the obtained supernatant. The supernatant was centrifuged for 10 min at 10,000 *g*, 4°C, which produced a mitochondrial pellet and cytosolic mitochondrion-free supernatant.

Mitochondrial protein fraction was obtained by sonicating mitochondrial pellets in ice-cold buffer II (50 mM Tris, 5 mM EDTA, 150 mM NaCl, and protease inhibitors). Homogenates were incubated on ice for 30 min and centrifuged for 15 min at 10,000 *g*, 4°C, and supernatants containing mitochondrial proteins were collected.

Protein concentration was measured using Bradford assay (Bio-Rad, Hercules, CA, USA) according to manufacturer's instructions.

### Western blot analysis

We separated 35 μg of protein by SDS-PAGE. After transfer to a polyvinylidene fluoride membrane (Millipore, Billerica, MA, USA), membranes were blocked with 5% nonfat dry milk in 0.1% Tween-PBS (TBST) and incubated overnight at 4°C with primary antibody in 5% bovine serum albumin/TBST [mouse anti-p53 (Cell Signaling, Danvers, MA, USA), mouse anti-cytochrome c (BD Biosciences, Franklin Lakes, NJ, USA), rabbit anti-cleaved caspase 3 (Cell Signaling), mouse anti-HSP 70 (Santa Cruz Biotechnology, Santa Cruz, CA, USA), mouse anti-cytochrome *c* oxidoreductase (COX-IV) (Invitrogen), mouse anti-histone H1b (Abcam, Cambridge, MA, USA)] followed by horseradish peroxidase (HRP)-conjugated secondary antibodies (goat anti-mouse IgG or goat anti-rabbit immunoglobulin G (IgG; Jackson ImmunoResearch, West Grove, PA, USA) (in 5% nonfat dry milk/TBST). HRP-conjugated mouse anti-β-actin (Sigma-Aldrich) was used for normalization of cytosolic samples. Blots were revealed using enhanced chemiluminescence Amersham ECL Western Blotting Detection Reagent (GE Healthcare, Little Chalfont, Buckinghamshire, UK) and visualized, captured, and analyzed using a LAS system and Image Quant software (GE Healthcare).

### Mitochondrial membrane potential measurement

DRG neurons were dissociated as described (Ma J. et al., 2014) and stained with 200 nM of tetramethylrhodamine methyl ester (TMRM; Molecular Probes, Eugene, OR, USA) for 45 min at 37°C. After washing with phosphate buffer saline, the fluorescence signal was detected with a BD Accuri C6 Flow Cytometer (BD Biosciences, San Jose, USA) at emission of FL2 585/40 nm. The TMRM was used in sub-quench mode as described by Nicholls (Rego et al., 2001), where a decrease in fluorescence intensity indicates reduced mitochondrial membrane potential. As a positive control cells were incubated with 10 μM carbonilcyanide p-triflouromethoxyphenylhydrazone (FCCP), a mitochondrial uncoupler, for 15 min.

### Transmission electron microscopy for mitochondrial morphology

Abnormalities in mitochondrial structure and function in peripheral sensory neurons are thought to contribute to neuronal damage and neuropathic pain induced by chemotherapeutics (Bennett et al., 2014). We collected DRG and sciatic nerve 3 weeks after completion of two cycles of cisplatin ± PFT-μ treatment. Tissues were fixed in 3% glutaraldehyde/2% paraformaldehyde in 0.1 M cacodylate buffer, treated with 0.1% buffered tannic acid, post fixed with 1% buffered osmium tetroxide, and stained *en bloc* with 1% uranyl acetate. After dehydration in increasing concentrations of ethanol, ultrathin sections were cut in a Leica Ultra cut microtome, mounted on mesh copper grids, stained with uranyl acetate, and lead citrate in a Leica EM Stainer, and examined in a JEM 1010 transmission electron microscope (JEOL, USA, Inc., Peabody, MA, USA) at an accelerating voltage of 80 kV. Digital images were obtained using AMT Imaging System (Advanced Microscopy Techniques Corp, Danvers, MA, USA). Mitochondria were scored as typical or atypical according to a system developed by Flatters and Bennett (2006) and modified slightly by us (Krukowski et al., 2015). Atypical mitochondria were identified by disrupted cristae, damaged double membrane, and changes in size (above 300 nm in diameter). For peripheral nerve axons, 10–14 mitochondria were scored per mouse, and for DRG tissue, 14–20 mitochondria were scored. We showed previously that mice treated with PFT-μ alone did not display any difference in mitochondrial morphology, compared with animals treated with vehicle alone (Krukowski et al., 2015).

### Mitochondrial bioenergetics assessment

To test the effect of cisplatin on mitochondrial function, we used dissociated primary DRG cells and freshly isolated tibial nerves from mice treated with two cycles of cisplatin and displaying symptoms of peripheral neuropathy.

Immediately after collection of lumbar (L3-L6) DRG from cisplatin ± PFT-μ-treated and control mice and removal of connective tissue, ganglia were dissociated, and an enriched neuronal fraction was obtained as described (Ma J. et al., 2014). Cells were plated in polyornithine (conc. 0.01%, o/n)/laminin (2 μg/mL, 3–4 h)-coated XF24 plates (Seahorse Bioscience, Santa Clara, CA) at about 1,000 cells/well in Ham's F10 medium containing N2 supplement without insulin (Invitrogen). After overnight incubation, cells were washed with Seahorse Assay Media (pH 7.4), and placed in 500 μL of Assay Media (containing 5.5 mM glucose, 0.5 mM sodium pyruvate, and 1 mM glutamine) at 37°C for 1 h.

Tibial nerves (from the splitting of the sciatic nerve to immediately above the ankle) were dissected from cisplatin ± PFT-μ-treated and control mice and placed into an islet capture XF24 microplate (Seahorse Bioscience) in 500 μL Assay Media at 37°C for 2 h. Nerve tissues were sequentially exposed to oligomycin (12 μM), FCCP (20 μM), and Rotenone/Antimycin A (20 μM).

Oxygen consumption rate (OCR) was analyzed using the XF^e^24 Extracellular Flux Analyzer (Seahorse Bioscience). After assessing the basal OCR, we measured the effect of the following inhibitors of respiratory chain components: (1) oligomycin (2 μM), an ATP synthase inhibitor, which leads to a decrease in OCR that represents the portion of basal OCR coupled to ATP synthesis; (2) carbonilcyanide p-triflouromethoxyphenylhydrazone (FCCP) (4 μM), a protonophore that dissipates the proton gradient across the inner mitochondrial membrane, allowing measurement of the maximal respiratory capacity; and (3) a combination of rotenone (a complex I inhibitor) and antimycin A (a complex III inhibitor) (2 μM), which shuts down the mitochondrial respiration and enables calculation of non-mitochondrial respiration. Dissociated DRG were measured in an assay cycle of 3 min mix, 2 min wait, and 3 min measure, repeated 3 times. Nerve tissues were measured in an assay cycle of 3 min mix, 3 min wait, and 4 min measure, repeated 4 times to capture rates at baseline and after each drug injection.

OCR data were normalized to total protein levels as assessed after completion of OCR measurements.

### Behavioral tests

Mechanical allodynia was assessed with von Frey hairs, using the up-and-down method (Chaplan et al., 1994) as we described previously (Wang et al., 2013; Singhmar et al., 2016). Animals were placed in transparent plastic cages (5 × 10 × 13 cm^3^) on a metal mesh floor. A series of graded-strength von Frey hairs (0.02, 0.07, 0.16, 0.4, 0.6, 1.0, and 1.4 g) (Stoelting, Wood Dale, IL, USA) was applied to the mid-plantar surface of hind paw, starting with the 0.16 g hair. The force was decreased after a positive response (clear paw withdrawal or shaking) or increased after a negative response. Starting with the first positive response, we recorded the force needed to elicit five additional responses, and the 50% withdrawal threshold was calculated (Chaplan et al., 1994).

In patients, CIPN comprises not only increased sensitivity to painful stimulation, but also a loss of sensory function or hypoesthesia (Cleeland et al., 2010; Wolf et al., 2012). We have recently shown in a mouse model that numbness of the paw as a result of cisplatin treatment can be detected using the adhesive-removal test (Mao-Ying et al., 2014). This test measures the latency to first response to an adhesive patch placed on the hind paw. Briefly, we placed a round adhesive patch (3/15” Teeny Touch-Spots, USA Scientific INC, Ocala, FL, USA) on the plantar surface of the mouse hind paw and then placed the animal in a 10 × 20 × 13 cm^3^ enclosure with transparent walls. The time until the mouse displayed the first behavioral response to the patch as identified by shaking its paw or bringing the paw to its mouth was recorded. A longer latency to respond to the patch in cisplatin-treated mice vs. untreated controls was considered to be an indication of sensory deficit or numbness.

### Intra-epidermal nerve fiber density

CIPN is associated with a reduction in intra-epidermal nerve fiber (IENF) density in the plantar surface of the hind paws of rodents (Mao-Ying et al., 2014; Krukowski et al., 2015) and in patients treated for cancer (Jin et al., 2008; Boyette-Davis and Dougherty, 2011). To investigate whether PFT-μ protects against the cisplatin-induced loss of IENFs, we analyzed the density of fibers entering the epidermis in the glabrous skin of the hind footpad.

Tissues were obtained 5 weeks after the start of treatment, as previously described (Mao-Ying et al., 2014; Krukowski et al., 2015). Plantar surface samples of the hind paw were fixed in Zamboni's reagent, transferred to 20% sucrose, and frozen in 10% sucrose/optimal cutting temperature compound. Sections (25 μm) were blocked in 5% donkey serum/0.3% Triton X-100 in 0.1 M phosphate-buffered saline (PBS), stained for the pan-neuronal marker protein gene product (PGP) 9.5 (anti-rabbit; AbD Serotec, Raleigh, NC, USA) and for Collagen IV (anti-goat, Southern Biotech, Birmingham, AL, USA) to facilitate orientation, followed by Alexa-594-donkey anti-rabbit (1:500; Life Technologies, Carlsbad, CA, USA), and Alexa-488-donkey anti-goat (1:500; Invitrogen, Carlsbad, CA, USA). Three random sections from each paw (*n* = 6–8 paws/group) were analyzed using a Leica CTR 6500 confocal microscope (Leica Microsystems, Buffalo Grove, IL, USA). IENF density was counted using 40 × objective (Mao-Ying et al., 2014).

### Statistical analysis

Data are expressed as a mean ± standard error of the mean. Statistical analysis of the differences between multiple groups was performed by using one-way or two-way analysis of variance (ANOVA), with Tukey's post-test (Instat; GraphPad, San Diego, CA, USA); *P* < 0.05 was considered statistically significant.

## Results

### Cisplatin-induced mitochondrial p53 accumulation and mitochondrial membrane depolarization

**Figure 1:**
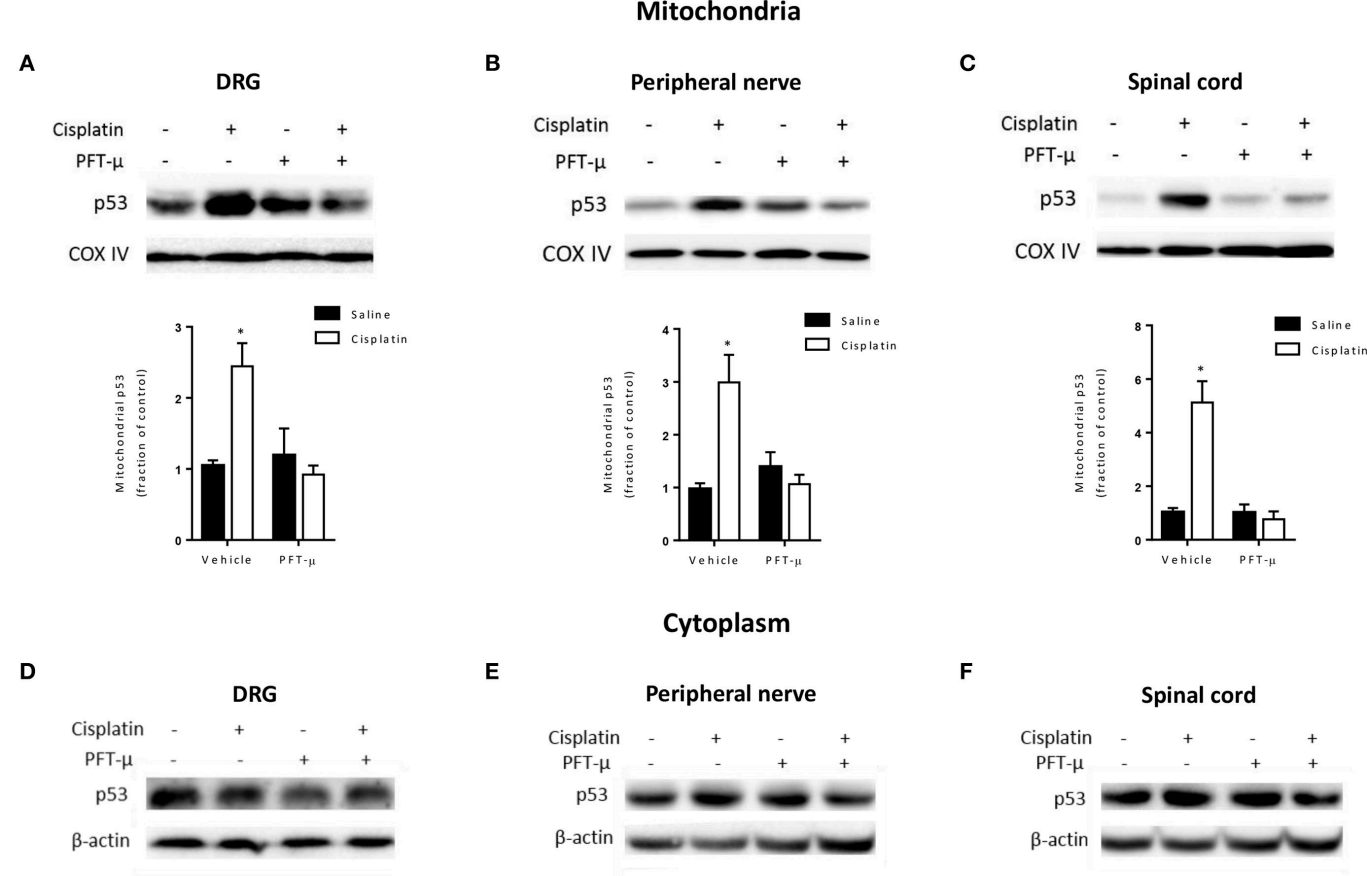
**PFT-μ prevents cisplatin-induced mitochondrial p53 accumulation**. Mice were treated with pifithrin-μ (PFT-μ) (8 mg/kg) or vehicle 1 h prior to a single intraperitoneal (i.p.) injection of cisplatin (2.3 mg/kg) or vehicle. Dorsal root ganglion (DRG), sciatic nerve, and lumbar spinal cord were collected 4 h later. Cytosolic fractions and mitochondria-enriched fractions were analyzed by Western blotting. **(A–C)** Western blot analysis of mitochondrial p53. Insets show representative Western blots with the mitochondrial protein cytochrome c oxidase subunit IV (COX IV) as a loading control. Bar graphs represent mean ± standard error of the mean (SEM) for a representative experiment out of 3 independent experiments in which 3 independent samples, each consisting of DRG from 2 mice, spinal cord from 3 mice, or nerve from 3 mice were used. Two-way analysis of variance (ANOVA) detected a significant (*P* < 0.05) cisplatin × PFT-μ interaction for all tissues [*F*_(1, 8)_ = 15.35 for DRG; *F*_(1, 8)_ = 15.87 for nerve; *F*_(1, 8)_ = 25.14 for spinal cord]. Tukey *post-hoc* test: ^*^P < 0.05 cisplatin + vehicle vs. cisplatin + PFT-μ. **(D–F)** Western blot analysis of p53 in cytosolic fractions from the same tissue samples. Insets show representative Western blots with the cytosolic β-actin as a loading control.

We recently showed that cisplatin treatment induces a rapid and transient increase in mitochondrial p53 in the brain (Chiu et al., 2017). To determine whether cisplatin also induces mitochondrial p53 accumulation in the peripheral nervous system, we injected a single dose of 2.3 mg/kg cisplatin and prepared mitochondrial, cytosolic and nuclear fractions from lumbar DRG, peripheral nerve, and lumbar spinal cord. The purity of the fractions was confirmed by Western blot analysis for COX IV, β-actin and histone H1b (Supplementary Figure 1). Immunoblot analysis revealed a significant increase in p53 protein levels in mitochondrial fractions of lumbar DRG, sciatic nerve, and lumbar spinal cord at 4 h after cisplatin injection as compared with vehicle treatment (Figures 1A–C). Cisplatin did not induce a detectable increase in p53 in cytosolic fractions (Figures 1D–F) or nucleus (Supplementary Figure 1 and data not shown). The mitochondrial p53 level was lower at 24 h after cisplatin and had returned to baseline levels 72 h after a single injection of cisplatin (Supplementary Figure 2).

The cisplatin-induced mitochondrial p53 accumulation in DRG, sciatic nerve, and lumbar spinal cord was prevented by co-administration of the small compound PFT-μ, an inhibitor of mitochondrial p53 accumulation (Figures 1A–C).

**Figure 2:**
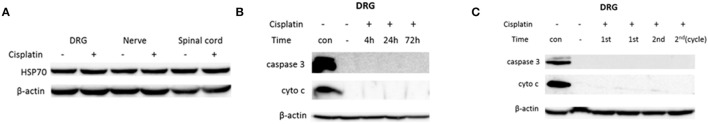
**Cisplatin does not induce markers of apoptosis in neuronal tissue**. Mice were treated with PFT-μ (8 mg/kg) or vehicle 1 h prior to an i.p. injection of cisplatin (2.3 mg/kg) or vehicle. Dorsal root ganglion (DRG), sciatic nerve, and lumbar spinal cord were collected at various time points. Whole-cell lysates or cytosolic fractions were analyzed by Western blotting. **(A)** Western blot analysis of heat-shock protein 70 (HSP70) on whole cell lysate from tissues collected 4 h after single cisplatin treatment. **(B–C)** Western blot analysis of apoptotic markers: cytosolic cytochrome c and cleaved caspase 3: of DRG tissues collected 4, 24, and 72 h after a single dose of cisplatin (2.3 mg/kg) **(B)**, and DRG collected on a last day of 1st and 2nd cycle of 5 daily cisplatin injections per cycle (the final cumulative dose of cisplatin was 11.5 and 23 mg/kg, respectively) **(C)**. “Con” designates positive controls: whole cell lysate of H_2_O_2_-treated N2A cells for cleaved caspase 3, and lysate of a mitochondrial fraction for cytochrome c. Data of one representative experiments out of three with 3 mice per group for each measure are presented. β-actin was used as a loading control.

There is evidence that PFT-μ may impact cellular function via the HSP70 complex; therefore we tested whether cisplatin has an effect on the level of this protein. The results in Figure 2A show that cisplatin did not alter HSP70 protein expression level, reducing the likelihood that PFT-μ acts in our model by targeting HSP70 (Figure 2A).

Next, we determined whether the cisplatin-induced increase in mitochondrial p53 resulted in activation of apoptotic pathways. The immunoblot analysis of cytosolic fractions from DRG depicted in Figures 2B,C demonstrates no detectable changes in cytochrome c or cleaved caspase 3 over a time period of 72 h after a single dose of cisplatin (Figure 2B). To induce CIPN, mice are treated with two cycles of 5 daily doses of cisplatin (2.3 mg/kg/day i.p.) followed by 5 days of rest. Therefore, we also examined the effect of the first and second cycle of cisplatin treatment on apoptotic pathways. As is shown in Figure 2C, we did not detect changes in activated caspase 3 nor the presence of cytosolic cytochrome c in DRG samples after completion of the first and second cycle of daily single cisplatin injections.

**Figure 3:**
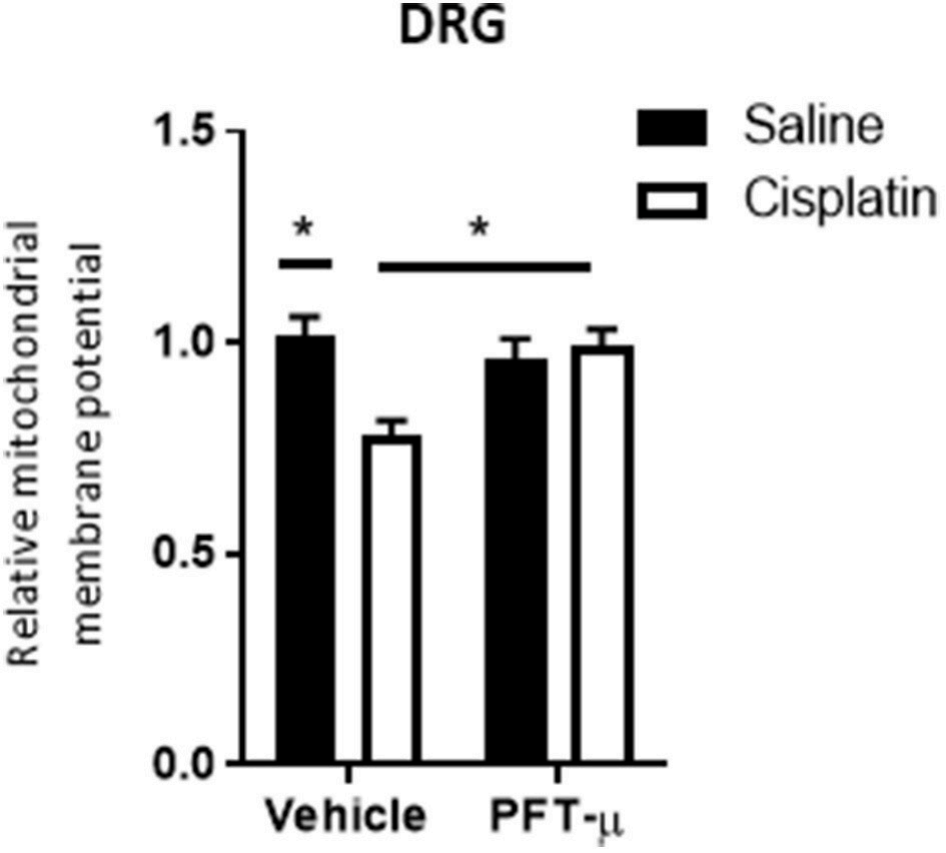
**PFT-μ prevents the cisplatin-induced decrease in mitochondrial membrane potential**. Flow cytometric analysis of dorsal root ganglion (DRG) neurons collected 4 h after single cisplatin (2.3 mg/kg) injection ± pre-treatment with PFT-μ (8 mg/kg). DRG neurons were stained with the membrane potential-sensitive dye tetramethylrhodamine methyl ester (TMRM) and analyzed by flow cytometry. Bar graphs represent mean ± standard error of the mean (SEM) of 3 mice per group. Two-way analysis of variance (ANOVA) detected a significant (*P* < 0.05) group × group interaction [*F*_(1, 8)_ = 9.63]. Tukey *post-hoc* test: ^*^P < 0.05 cisplatin + vehicle vs. cisplatin + PFT-μ.

*In vitro* studies have indicated that the accumulation of p53 at mitochondria precedes changes in mitochondrial membrane potential (Marchenko et al., 2000). To assess whether accumulation of mitochondrial p53 by cisplatin led to an acute change in mitochondrial membrane potential, we stained DRG neurons, obtained 4 h after single administration of cisplatin, with the membrane potential-sensitive dye tetramethylrhodamine methyl ester (TMRM). Flow cytometric analysis demonstrated that cisplatin-treatment resulted in a reduction in the mitochondrial membrane potential (Figure 3). Co-administration of PFT-μ prevented this cisplatin-induced mitochondrial membrane depolarization in DRG neurons (TMRM fluorescence intensity did not change as compared to vehicle treatment) (Figure 3). FCCP was used as a positive control for mitochondrial membrane depolarization, and resulted in a 25 ± 5% (*n* = 3) decrease of TMRM fluorescence intensity.

### Role of mitochondrial p53 accumulation in the cisplatin-induced abnormalities in mitochondrial morphology and bioenergetics

There is evidence from studies in male rodents that CIPN is associated with persistent mitochondrial abnormalities (Xiao et al., 2011, 2012). We investigated whether cisplatin-induced peripheral neuropathy in females is associated with mitochondrial abnormalities and determined the potential protective effect of PFT-μ. Mice were treated with two cycles of cisplatin ± PFT-μ. This treatment schedule induces peripheral neuropathy (Mao-Ying et al., 2014). PFT-μ (8 mg/kg, i.p.) was administered daily from 1 day before the first injection of cisplatin until the last day of cisplatin treatment in both cycles, and tissues were collected 3 weeks after completion of cisplatin treatment.

**Figure 4:**
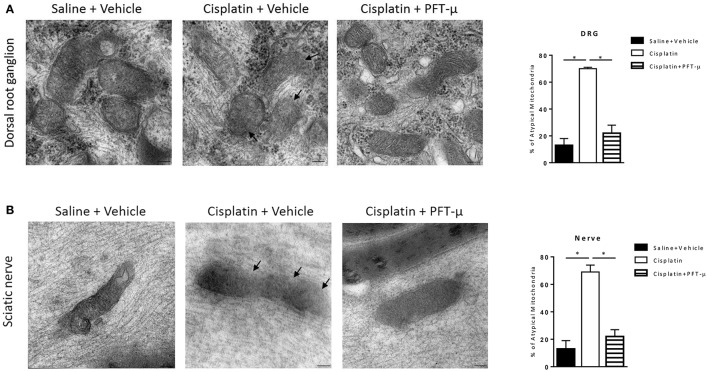
**PFT-μ prevents cisplatin-induced abnormalities in mitochondrial morphology**. Transmission electron microscopy images of mitochondria in dorsal root ganglion (DRG) neurons **(A)** and sciatic nerve **(B)** isolated from mice (*n* = 3 mice per group) treated with cisplatin (2 cycles of 2.3 mg/kg/day for 5 days followed by 5 days rest, resulting in a cumulative dose of 23 mg/kg) + vehicle, cisplatin + pifithrin-μ (PFT-μ) (2 cycles of 6 daily injections of 8 mg/kg i.p starting 1 day before cisplatin), or saline + vehicle. Samples were collected 5 weeks after start of cisplatin treatment. Representative micrographs (magnification × 75.000), and bar graphs of a mean % of atypical mitochondria ± standard error of the mean (SEM). One-way analysis of variance (ANOVA) revealed significant differences between groups: *P* < 0.0001 for DRG [*F*_(2, 6)_ = 93.43] and nerve [*F*_(2, 6)_ = 62.66]; Tukey *post-hoc* test: ^*^P < 0.001 for cisplatin + vehicle vs. cisplatin + PFT-μ and saline + vehicle. Arrows denote disrupted double membrane and disorganized cristae.

The transmission electron microscopy images depicted in Figure 4 demonstrate that treatment with cisplatin resulted in abnormal mitochondrial morphology in DRG and sciatic nerve. Mitochondria from cisplatin-treated mice displayed disorganized cristae and disrupted double membranes (Figures 4A,B middle image, denoted by arrows).

Co-administration of PFT-μ prevented the structural changes in mitochondria in the DRG (Figure 4A) and sciatic nerve (Figure 4B), and the frequency of abnormal mitochondria was similar in cisplatin + PFT-μ and vehicle-treated mice.

Next, we performed *ex vivo* analysis of mitochondrial function in dissociated DRG neurons and tibial nerves. We measured the oxygen consumption rate (OCR) after treating DRG neurons or tibial nerve sequentially with oligomycin, FCCP, and a mix of rotenone and antimycin A. Oligomycin inhibits ATP synthase and the oligomycin-induced decrease in OCR represents the mitochondrial respiration associated with cellular ATP production. The mitochondrial uncoupler FCCP removes the pH gradient and enables maximal rates of electron transport, to quantify the maximal consumption of oxygen by complex IV. The combination of rotenone and antimycin A blocks respiratory electron flux at complex I and III, thereby shutting down mitochondrial respiration to allow quantification of non-mitochondrial respiration.

**Figure 5:**
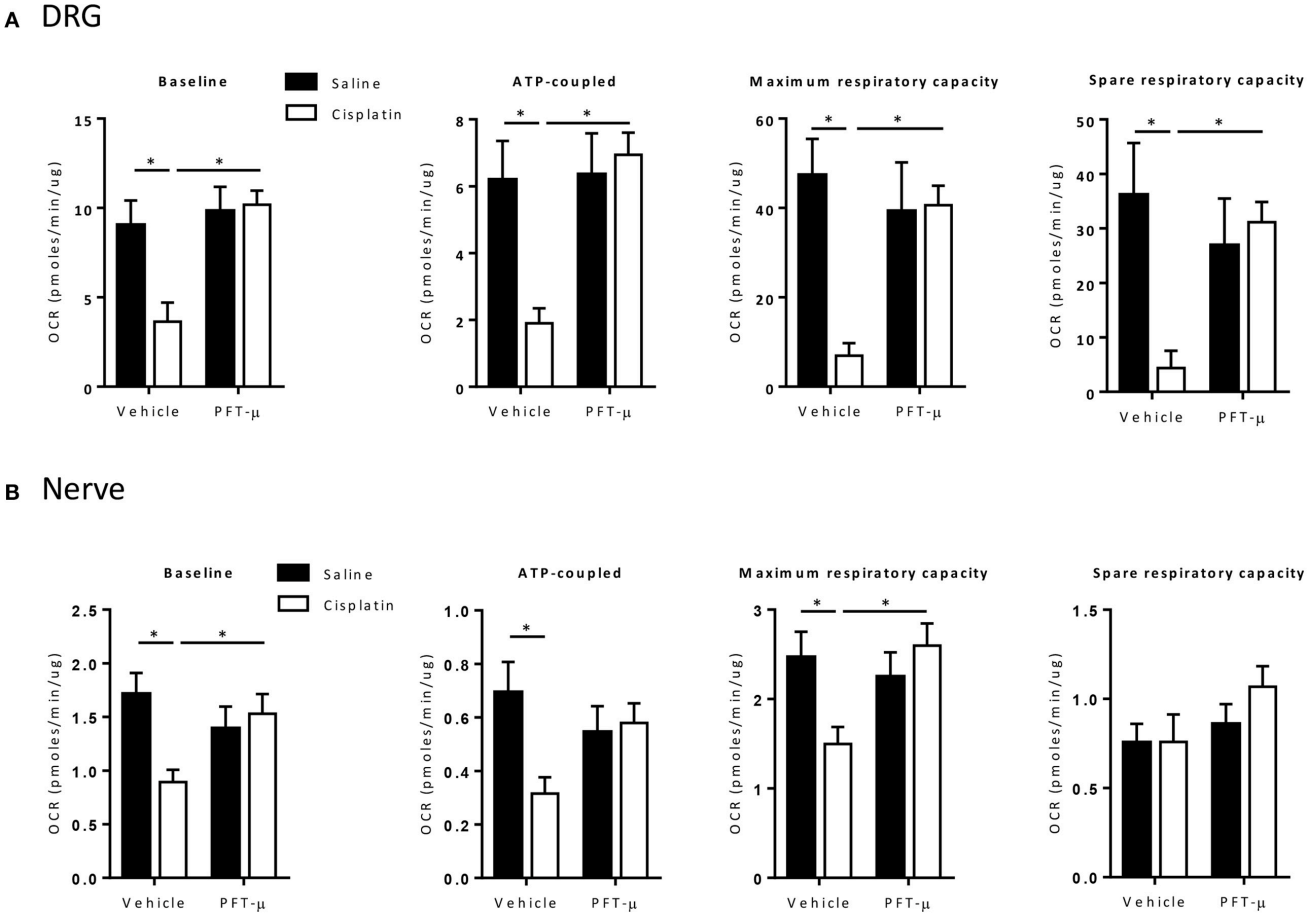
**PFT-μ protects against cisplatin-induced neuronal mitochondrial dysfunction**. Bioenergetic analysis of mitochondrial function of dissociated dorsal root ganglion (DRG) cells **(A)** and tibial nerve **(B)** collected 5 weeks after start of cisplatin ± pifithrin-μ (PFT-μ) treatment. Mice (*n* = 12 per group) were treated with cisplatin (2 cycles of 2.3 mg/kg/day for 5 days followed by 5 days rest, resulting in a cumulative dose of 23 mg/kg) + vehicle, cisplatin + pifithrin-μ (PFT-μ) (2 cycles of 6 daily injections of 8 mg/kg i.p starting 1 day before cisplatin), or saline + vehicle. The changes in the baseline oxygen consumption rate (OCR), and response to oligomycin (2 uM) (ATP synthesis), FCCP (carbonilcyanide p-triflouromethoxyphenylhydrazone) (4 uM) (maximum OCR), and rotenone/antimycin A (2 uM) (extra mitochondrial OCR) in dissociated DRG cells **(A)** and tibial nerve **(B)**. Bar graphs represent mean ± standard deviation (SD) of 12 mice per group. Two-way analysis of variance (ANOVA): group effect (*P* < 0.05) [*F*_(1, 42)_ = 9.71 for DRG, *F*_(1, 40)_ = 4.08 for nerve], treatment effect (*P* < 0.05) [*F*_(1, 42)_ = 11.80 for DRG, *F*_(1, 40)_ = 1.76 for nerve], and group × treatment interaction (*P* < 0.05) [*F*_(1, 42)_ = 7.49 for DRG, *F*_(1, 40)_ for 7.08 for nerve]; Tukey *post-hoc* test: ^*^P < 0.05 for cisplatin + vehicle vs. cisplatin + PFT-μ and saline + vehicle groups, as indicated on graphs.

DRG neurons and tibial nerves from cisplatin-treated mice showed a significant decline in total OCR, ATP-coupled OCR, and maximal respiratory capacity, indicating impairment of basal respiration and of the maximal electron transport activity. DRG neurons also showed a significant decline in spare respiratory capacity (SRC) (Figure 5). The decrease in SRC reflects the bioenergetics reserve that is available to respond to cellular energy demands.

Interestingly, the co-administration of PFT-μ prevented the cisplatin-induced impairment in mitochondrial function. PFT-μ alone did not affect mitochondrial function (Figure 5).

### Blocking mitochondrial p53 accumulation prevents CIPN

**Figure 6:**
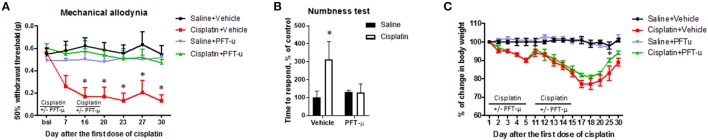
**Pifithrin-μ prevents mechanical allodynia and sensory dysfunction in a mouse model of chemotherapy-induced peripheral neuropathy**. Mice (*n* = 8 per group) were treated with cisplatin (2 cycles of 2.3 mg/kg/day for 5 days followed by 5 days rest, resulting in a cumulative dose of 23 mg/kg) + vehicle, cisplatin + pifithrin-μ (PFT-μ) (2 cycles of 6 daily injections of 8 mg/kg i.p starting 1 day before cisplatin), or saline + vehicle. **(A)** Mechanical allodynia was monitored over time with von Frey hairs. Data represent mean and SEM of 50% withdrawal threshold. Two-way repeated-measures analysis of variance (ANOVA) identified a group effect (*P* < 0.0001) [*F*_(3, 24)_ = 97.40], a time effect (*P* < 0.0001) [*F*_(6, 144)_ = 8.59], and a group × time interaction effect (*P* < 0.0001) [*F*_(18, 144)_ = 8.66]; Tukey *post-hoc* test: ^*^P < 0.0001 cisplatin + vehicle vs. all other groups. **(B)** Effect of cisplatin and PFT-μ on numbness (sensory function) as detected in the adhesive removal test; the time to display a behavioral response to an adhesive patch placed on the hind paw was recorded. Two-way ANOVA revealed a group effect (*P* < 0.005) [*F*_(1, 28)_ = 11.96], a treatment effect (*P* < 0.05) [*F*_(1, 28)_ = 11.75], and group × treatment interaction (*P* < 0.005) [*F*_(1, 28)_ = 4.69]; Tukey *post-hoc* test: ^*^P < 0.001 cisplatin + vehicle vs. all other groups. **(C)** Change in body weight after cisplatin and PFT-μ treatment. Two-way ANOVA, multiple comparisons: ^*^P < 0.01 between cisplatin + vehicle vs. cisplatin + PFT-μ on day 25 (marked with an asterisk).

To investigate the contribution of the early mitochondrial p53 accumulation to the development of cisplatin-induced peripheral neuropathy, we measured mechanical allodynia, peripheral sensory loss, and loss of intraepidermal nerve fibers. Mice were treated with two cycles of cisplatin ± PFT-μ (8 mg/kg, i.p.). Mechanical allodynia was monitored over time. Mice treated with cisplatin developed mechanical allodynia as shown by a decreased paw withdrawal threshold in the von Frey test (Figure 6A). Importantly, administration of PFT-μ together with cisplatin completely prevented the development of mechanical allodynia in response to cisplatin (Figure 6A). No differences in paw withdrawal threshold were observed between mice treated with vehicle alone, PFT-μ alone, or cisplatin + PFT-μ.

The effect of PFT-μ on the sensory loss that is induced by cisplatin was assessed in the adhesive-removal test at 16 days after completion of the two cycles of cisplatin. In this test an increase in the time to respond to an adhesive patch placed on the hind paw is used as an indication of sensory loss or numbness (Mao-Ying et al., 2014). The response-latency was significantly increased in cisplatin-treated mice. The results in Figure 6B show that PFT-μ prevented the loss of sensory function induced by cisplatin treatment. We already showed previously that the two cycles of cisplatin treatment do not affect spontaneous locomotor activity (Chiu et al., 2017) indicating that the increased response time in cisplatin-treated mice is not due to abnormal motility of the mice.

Cisplatin-treated mice decreased in body weight and PFT-μ had a slight protective effect in the second cycle. PFT-μ alone showed no effect on body weight (Figure 6C).

**Figure 7:**
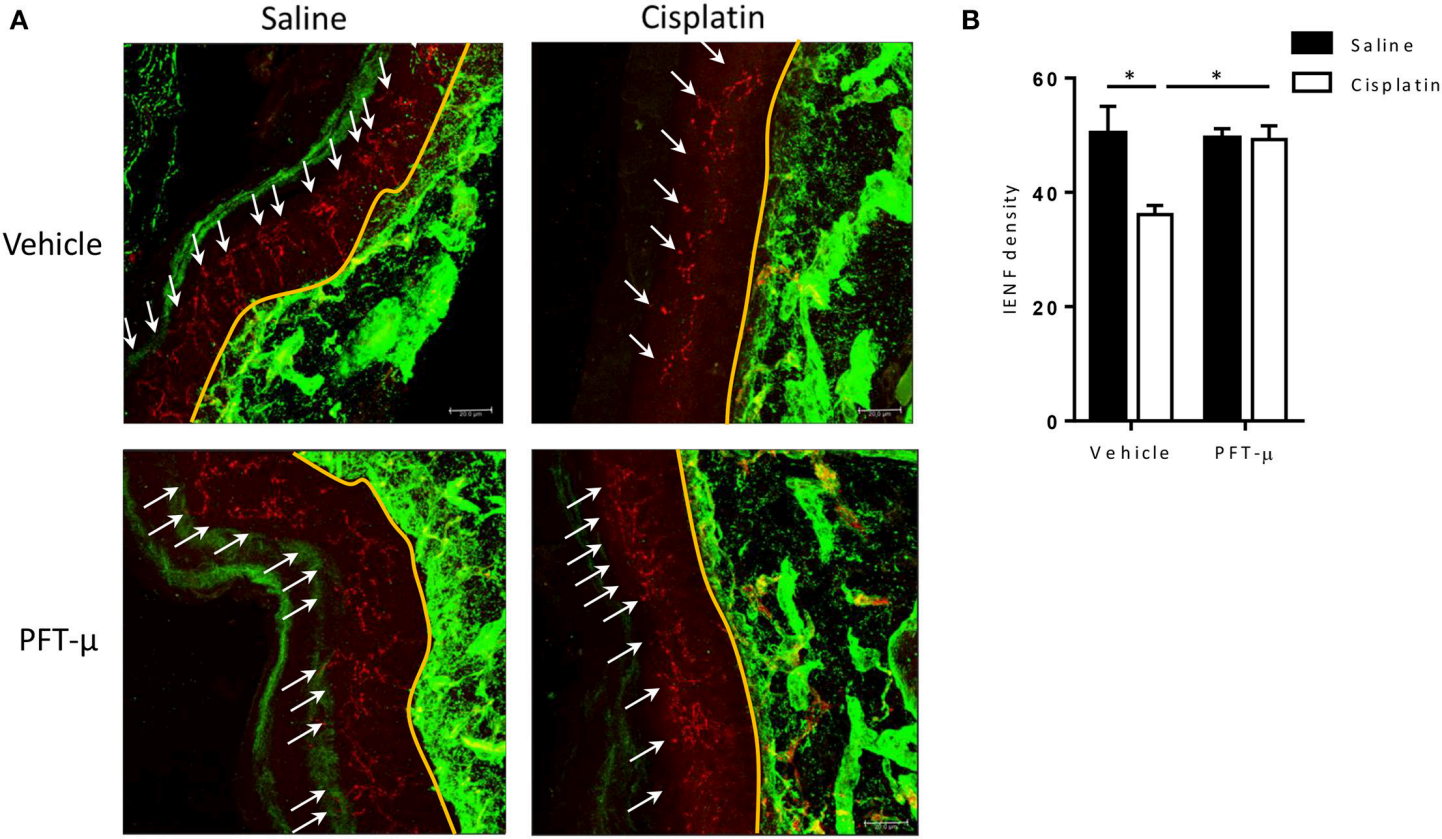
**PFT-μ prevents the cisplatin-induced reduction in intra-epidermal nerve fiber density**. Mice (*n* = 4 per group) were treated with cisplatin (2 cycles of 2.3 mg/kg/day for 5 days followed by 5 days rest, resulting in a cumulative dose of 23 mg/kg) + vehicle, cisplatin + pifithrin-μ (PFT-μ) (2 cycles of 6 daily injections of 8 mg/kg i.p starting 1 day before cisplatin), or saline + vehicle. Paw biopsies were collected 5 weeks after start of cisplatin ± PFT-μ, and were stained for IENFs using the pan-neuronal marker protein gene product (PGP) 9.5 (red) and collagen IV (green). **(A)** Immunofluorescence images of representative examples. **(B)** Quantification of intra-epidermal nerve fiber (IENF) density [IENF density = number of nerve fibers (shown in red) crossing the basement membrane (shown in green)/length of the basement membrane (mm)]; 8–10 sections of each mouse were quantified. Two-way analysis of variance (ANOVA) revealed a group effect (*P* < 0.05) [*F*_(1, 8)_ = 6.29] and a group × treatment interaction (*P* < 0.05) [*F*_(1, 8)_ = 5.86]; Tukey *post-hoc* test: ^*^P < 0.05 cisplatin + vehicle vs. all other groups. White arrows denote IENFs. Yellow landmark line shows the edge of the basement membrane. Scale bar = 21 μm.

We also analyzed the effect of cisplatin and PFT-μ on the density of fibers entering the epidermis in the glabrous skin of the hind footpad using the pan-neuronal marker PGP 9.5. In mice treated with cisplatin alone, we observed a significant reduction in IENF density (Figure 7). PFT-μ protected against IENF loss (Figure 7): IENF density in mice treated with cisplatin + PFT-μ was similar to the IENF density in saline-treated controls and in mice treated with PFT-μ alone.

## Discussion

Painful peripheral neuropathy as a result of chemotherapy is a condition that negatively impacts quality of life and cancer treatment outcomes (Coates et al., 1990; Hwang et al., 2013). The pathogenesis of CIPN is only partially understood and no preventive or curative intervention has been approved by the US Food and Drug Administration. The data reported here demonstrate that cisplatin administration results in a rapid (acute) increase in mitochondrial p53 in DRG, nerve, and spinal cord. We also show for the first time that cisplatin-induced mitochondrial p53 accumulation in the DRG results in a rapid (acute) decrease in mitochondrial membrane potential and initiates the development of persistent (chronic) mitochondrial functional and morphological abnormalities. On the basis of our findings, we propose that cisplatin induces a rapid and transient increase in mitochondrial p53 that is sufficient to initiate a mitochondrial damage pathway that leads to persistent mitochondrial functional and morphological abnormalities and the behavioral phenotype characteristic of CIPN. This model is supported by our finding that prevention of the acute increase in mitochondrial p53 by co-administration of the small molecule PFT-μ was sufficient to protect against the subsequent mitotoxicity in peripheral sensory neurons and spinal cord as well as against the mechanical allodynia, sensory loss, and reduction in IENF density that characterize CIPN. On the basis of these findings, we propose that prevention of p53 accumulation at neuronal mitochondria is an attractive therapeutic target. Since PFT-μ has anti-tumor activity as well, it may become an effective drug for the prevention of CIPN.

We show here for the first time that *in vivo* cisplatin induces an acute accumulation of p53 at the mitochondria of DRG neurons, peripheral nerve, and spinal cord. An *in vitro* study in HeLa cells (Li et al., 1999) has shown that mitochondrial p53 precedes a reduction in mitochondrial membrane potential. Here we demonstrate that *in vivo* the cisplatin–induced increase in mitochondrial p53 is associated with a rapid loss of mitochondrial membrane potential (Δψm) in DRG neurons. We also show that pre-treatment with the small compound PFT-μ prevents *both* the increase in mitochondrial p53 and the subsequent reduction in mitochondrial membrane potential in sensory neurons. These findings indicate that the increase in mitochondrial p53 in response to cisplatin causes the loss of mitochondrial membrane potential.

It has been shown that the chemotherapeutics paclitaxel and oxaliplatin induce structural damage in mitochondria in the sciatic nerve and DRG of male rats (Xiao et al., 2012). We show here that the same happens in DRG neurons and sciatic nerve from female mice in response to two cycles of cisplatin. Functionally, we demonstrate that the cisplatin-induced structural mitochondrial damage is associated with impaired bioenergetics; cisplatin treatment reduced baseline oxygen consumption, ATP-coupled respiration, and the maximal oxygen consumption rate in DRG and tibial nerve as measured 3 weeks after completion of cisplatin treatment. In the tibial nerve, baseline oxygen consumption and ATP-coupled respiration were also reduced, but we did not detect a change in maximal respiratory capacity. One possible explanation for the difference between DRG and nerve is that mitochondrial bioenergetics as measured in the DRG primarily reflect the activity of mitochondria in sensory neurons. In the tibial nerve, however, there is a contribution of mitochondria present in motor neurons as well as Schwann cells. Motor neurons are thought to accumulate less cisplatin than sensory neurons because the blood brain barrier protects them (Xiao et al., 2011). In addition, it is likely that there is preferential transport of still healthy mitochondria from DRG to nerve (Sajic et al., 2013), which may contribute to the smaller cisplatin-induced decrease in maximal respiratory capacity in the nerve as compared to DRG as well. Prevention of the mitochondrial accumulation of p53 by co-administration of PFT-μ protected against all of these long-term mitochondrial impairments in both DRG and nerve. On the basis of these data, we propose that rapid mitochondrial p53 accumulation results in changes in mitochondrial membrane potential and activation of a damage pathway that leads to long-lasting impairment in mitochondrial respiratory function and abnormalities in mitochondrial morphology.

The small compound PFT-μ was discovered during a screen for inhibitors of p53-dependent apoptosis that do not affect the transcriptional function of p53 (Strom et al., 2006). Beyond its important nuclear and transcriptional roles, the tumor-suppressor protein p53 is recognized to participate in the induction of apoptosis when localized to mitochondria (Baptiste and Prives, 2004; Green and Kroemer, 2009; Mossalam et al., 2012; Tendler et al., 2013). Cellular stress induces association of p53 with mitochondria via interactions with members of Bcl-2 family proteins (Geng et al., 2010; Han et al., 2010; Mossalam et al., 2012), and thereby p53 is thought to facilitate mitochondrial outer membrane permeabilization, leading to cytochrome c release into the cytosol, and caspase-3–mediated apoptosis (Vaseva and Moll, 2009). Strom et al. (2006) showed that PFT-μ protected against p53-mediated apoptosis caused by radiation of thymocytes. In addition to its protective effect in models of CIPN described in the present study and our previous work (Krukowski et al., 2015), we have shown that PFT-μ protects against neonatal hypoxic–ischemic (HI) brain damage (Nijboer et al., 2011). In this model, PFT-μ administration prevented cognitive impairment and sensorimotor dysfunction associated with HI-induced brain apoptosis and structural damage (Nijboer et al., 2011). In addition, results from a recent study in a Parkinson's mouse model showed that PFT-μ prevents 1-methyl-4-phenyl-1, 2, 3, 6-thetrahydropyridine (MPTP)-induced neurotoxicity by inhibiting mitochondrial p53/Bcl-XL interactions, impaired mitochondrial transmembrane potential, and cytosolic cytochrome c release (Shin et al., 2016). Several studies using dissociated DRG neurons demonstrate that *ex vivo* incubation with cisplatin activates an apoptotic pathway as shown by Tunel staining, along with cytochrome c release and increase of reactive oxygen species production (McDonald and Windebank, 2002; Jiang et al., 2008; Melli et al., 2008; Florea and Busselberg, 2011). In contrast to the results obtained in these *in vitro* studies, in our *in vivo* model of cisplatin-induced neuropathy, we did not observe cytochrome c release to the cytoplasm or activation of caspase-3. These findings indicate that, *in vivo*, cisplatin-induced CIPN requires mitochondrial p53 accumulation but is not associated with detectable neuronal apoptosis in the periphery. These findings are consistent with those of Li et al. (1999) who showed in an *in vitro* model in HeLa cells that mitochondrial accumulation of p53 lead to abnormal mitochondrial membrane potential without triggering the entire apoptotic cascade.

We show here that PFT-μ prevents CIPN induced by cisplatin in female mice. We recently demonstrated that PFT-μ also protects against the cognitive deficits induced by cisplatin treatment in male mice (Chiu et al., 2017). The results from that study demonstrated that a single injection of cisplatin rapidly induces mitochondrial accumulation of p53 in the brain as well. Co-administration of PFT-μ not only prevented this mitochondrial p53 accumulation but also the mitochondrial dysfunction, morphological abnormalities and the cognitive deficits induced by cisplatin. Similar to what we show here for the peripheral nervous system, we did not detect evidence for cisplatin-induced activation of apoptotic pathways in the brain. These findings indicate that the same mechanisms of cisplatin-induced neurotoxicity are operative in males and females and in peripheral and central nervous system. Interestingly, although we did detect cisplatin-induced functional deficits in mitochondrial bioenergetics in the brain synaptosomes as well as in peripheral neurons (DRG), the nature of the deficits was different. We show here that in the peripheral nervous system oxygen consumption at baseline, ATP production-related oxygen consumption, and maximal respiratory capacity are all reduced in response to cisplatin treatment. In contrast, in the brain, only the maximal, and spare respiratory capacity were reduced, while baseline and ATP production-related oxygen consumption were not affected. This difference may be due to exposure of the brain to a lower dose of cisplatin because of limited penetration of the blood brain barrier or to a potentially larger mitochondrial reserve in the brain. Nevertheless, both brain and peripheral mitochondrial damage was prevented by PFT-μ.

Our present findings and previous work (Krukowski et al., 2015) indicate that the mechanism of action of cisplatin-induced peripheral neuropathy might have more general validity for CIPN. We recently published that PFT-μ prevented paclitaxel-induced mechanical allodynia, IENF loss, and morphological mitochondrial abnormalities in sensory neurons in mice (Krukowski et al., 2015). Paclitaxel and cisplatin have a different mode of action since paclitaxel acts by stabilizing the microtubules polymers while cisplatin acts via formation of DNA adducts and thus inhibition of DNA replication. Nevertheless, PFT-μ is protective in both models of CIPN. Although we did not investigate whether paclitaxel increases mitochondrial p53 (Krukowski et al., 2015), we would like to suggest that acute neuronal mitochondrial accumulation of p53 is a shared pathway leading to neuronal mitochondrial dysfunction and contributing to the development of CIPN.

Disesthesia or numbness is a profoundly negative and debilitating neurotoxic symptom experienced by many cancer patients and survivors (Cleeland et al., 2010). Notably, in our CIPN mouse model cisplatin has no effect on the spontaneous locomotor activity of animals (Chiu et al., 2017), what indicates that a latency measurement in the numbness test is not influenced by motility of the animals. Here we report for the first time that blocking p53 accumulation with a small molecule PFT-μ, a “mitochondrial protectant,” effectively protects against cisplatin-induced numbness. The mechanism of peripheral numbness has not been clarified either, but it has been suggested that retraction of distal nerve endings may contribute to this neurotoxic symptom (Boyette-Davis et al., 2013). Our current discovery that blocking p53 accumulation by PFT-μ prevents peripheral cisplatin-induced numbness, strongly suggests that local peripheral mitochondrial dysfunction does not only lead to pain but also plays a crucial role in the establishment of peripheral numbness. This is also supported by the finding that cisplatin induced significant damage to the epidermal innervation, while co-administration of PFT-μ prevented the loss of innervation.

Emerging data suggest that PFT-μ may act as an anticancer agent (Kaiser et al., 2011; Monma et al., 2013; Sekihara et al., 2013; Ma L. et al., 2014). For example, in pancreatic cell lines, PFT-μ significantly decreased the viability and colony-forming ability of cancer cells, increased annexin V(+) cells, and eventually arrested cancer cell growth (Monma et al., 2013). In line with these findings, we have shown that PFT-μ enhanced paclitaxel-induced death of the human ovarian tumor cells (HeyA8) (Krukowski et al., 2015). It has been suggested that the antitumor effects of PFT-μ are mediated via inhibition of an interaction of HSP70 with lysosome-associated membrane protein 2, leading to dysregulation of autophagy and enhanced tumor cell death (Leu et al., 2009). We recently showed that PFT-μ does not inhibit the reduction in tumor size in a syngenic human papillomavirus mouse model (C57BL/6 mice) treated with a combination of chemotherapy and irradiation (Chiu et al., 2017).

Taken together, our data suggest that the cisplatin-induced mitochondrial p53 accumulation in peripheral neurons initiates a cascade of events leading to functional mitochondrial impairments and thereby to histopathological, and behavioral deficits in our CIPN mouse model. Our results highlight the potential of PFT-μ as a therapeutic strategy to manipulate the mitochondrial p53 pathway to confer protection against cisplatin-induced neurotoxicity.

## Author contributions

MM, designed research, performed research, analyzed data, wrote the manuscript; JM, performed research, analyzed data, edited manuscript; KK, contributed to work design, analyzed data, edited manuscript; AK, designed research, supervised the project, wrote the manuscript; CH, designed research, supervised the project, wrote the manuscript.

## Funding

This study was supported by NIH R21 CA1837365 and NIH R01 CA193522.

### Conflict of interest statement

The authors declare that the research was conducted in the absence of any commercial or financial relationships that could be construed as a potential conflict of interest.

## Supplementary material

Supplementary Figure1 **Western blot analysis of dorsal root ganglion (DRG) tissue collected 4 h after a single dose of cisplatin**. Confirmation of fractionation quality; predominance of β-actin in the cytoplasm (cytopl), exclusive presence of cytochrome c oxidase subunit IV (COX IV) in the mitochondria (mitoch), and histone H1 in nuclear (nuclear) preparations. The results also show that cisplatin increased mitochondrial p53 without changes in nuclear or cytosolic p53.

Supplementary Figure2 **Mice were injected with a single dose of cisplatin (2.3 mg/kg) or vehicle, and dorsal root ganglion (DRG) tissue was collected 4, 24, and 72 h later**. Western blot analysis of mitochondrial p53; the mitochondrial protein cytochrome c oxidase subunit IV (COX IV) was used as a loading control. Representative example out of 3 independent experiments. Each lane represents tissue from 2 mice.
