# Supplementary figures and images for: Inhibition of Mitochondrial p53 Accumulation by PFT-μ Prevents Cisplatin-Induced Peripheral Neuropathy
Source: Front Mol Neurosci. 2017 Apr 18;10:108. doi: 10.3389/fnmol.2017.00108 (PMC5394177; doi:10.3389/fnmol.2017.00108)

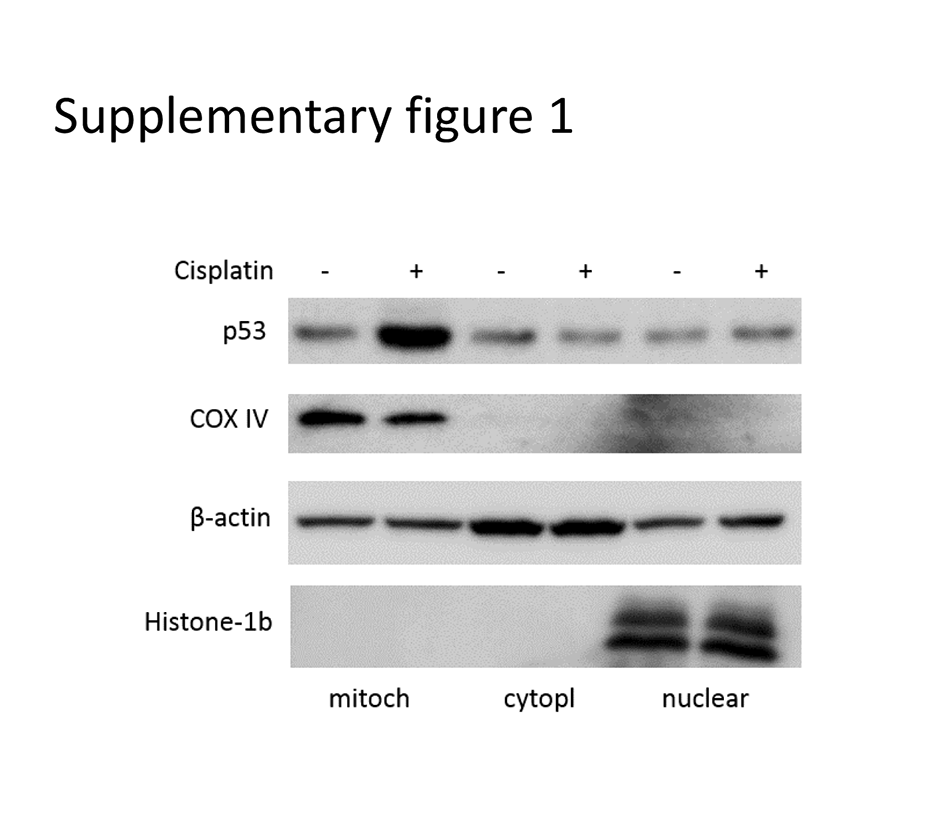

Supplement: Supplementary Figure1 — Western blot analysis of dorsal root ganglion (DRG) tissue collected 4 h after a single dose of cisplatin. Confirmation of fractionation quality; predominance of β-actin in the cytoplasm (cytopl), exclusive presence of cytochrome c oxidase subunit IV (COX IV) in the mitochondria (mitoch), and histone H1 in nuclear (nuclear) preparations. The results also show that cisplatin increased mitochondrial p53 without changes in nuclear or cytosolic p53. [file Image1.TIF]

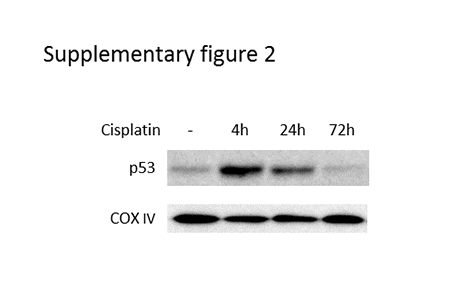

Supplement: Supplementary Figure2 — Mice were injected with a single dose of cisplatin (2.3 mg/kg) or vehicle, and dorsal root ganglion (DRG) tissue was collected 4, 24, and 72 h later. Western blot analysis of mitochondrial p53; the mitochondrial protein cytochrome c oxidase subunit IV (COX IV) was used as a loading control. Representative example out of 3 independent experiments. Each lane represents tissue from 2 mice. [file Image2.TIF]
